# Subjective health complaints, functional ability, fear avoidance beliefs, and days on sickness benefits after work rehabilitation – a mediation model

**DOI:** 10.1186/s12891-016-1084-x

**Published:** 2016-05-23

**Authors:** Irene Øyeflaten, Jon Opsahl, Hege R. Eriksen, Tore Norendal Braathen, Stein Atle Lie, Søren Brage, Camilla M. Ihlebæk, Kyrre Breivik

**Affiliations:** National Centre for Occupational Rehabilitation, Haddlandsvegen 20, NO-3864 Rauland, Norway; Uni Research Health, Bergen, Norway; Department of Sport and Physical Activity, Bergen University College, Bergen, Norway; Faculty of Health and Social Studies, Telemark University College, Porsgrunn, Norway; Department of Clinical Dentistry, University of Bergen, Bergen, Norway; The Directorate of Labour and Welfare, Oslo, Norway; Section of Public Health Science, ILP, Norwegian University of Life Sciences, Ås, Norway

**Keywords:** Sickness absence, Sick leave, Functional ability, Subjective health complaints, Musculoskeletal diseases, Mental disorders, Fear avoidance beliefs, Rehabilitation, Prognostic factors, Return to work

## Abstract

**Background:**

Long-term sick leave and withdrawal from working life is a concern in western countries. In Norway, comprehensive inpatient work rehabilitation may be offered to sick listed individuals at risk of long-term absence from work. Knowledge about prognostic factors for work outcomes after long-term sick leave and work rehabilitation is still limited. The aim of this study was to test a mediation model for various hypothesized biopsychosocial predictors of continued sick leave after inpatient work rehabilitation.

**Methods:**

One thousand one hundred fifty-five participants on long-term sick leave from eight different work rehabilitation clinics answered comprehensive questionnaires at arrival to the clinic, and were followed with official register data on sickness benefits for 3 years. Structural equation models were conducted, with days on sickness benefits after work rehabilitation as the outcome.

**Results:**

Fear avoidance beliefs for work mediated the relation between both musculoskeletal complaints and education on days on sickness benefits after work rehabilitation. The relation between musculoskeletal complaints and fear avoidance beliefs for work was furthermore fully mediated by poor physical function. Previous sick leave had a strong independent effect on continued sick leave after work rehabilitation. Fear avoidance beliefs for work did not mediate the small effect of pseudoneurological complaints on continued sick leave. Poor coping/interaction ability was neither related to continued sick leave nor fear avoidance beliefs for work.

**Conclusions:**

The mediation model was partly supported by the data, and provides some possible new insight into how fear avoidance beliefs for work and functional ability may intervene with subjective health complaints and days on sickness benefits after work rehabilitation.

## Background

The prevalence of long-term sick leave and disability pension is undesirably high in many industrialized countries [1]. To address some of these challenges, the Norwegian health service offers comprehensive inpatient work rehabilitation (WR) to individuals on long-term sick leave. The goal of WR is to assist individuals back to work through comprehensive programs where physical activity, cognitive behavioral modification, and cooperation with involved stakeholders are important elements. This is done within the frame of an interdisciplinary biopsychosocial rehabilitation model [2–4].

There are large individual differences in the process of returning to work (RTW) after long-term sick leave [5–7]. Previous research suggests that this process may be influenced by multifaceted biopsychosocial factors [8, 9]. In Norway, the most common diagnoses related to long-term sick leave and disability pension are musculoskeletal complaints and mild or moderate mental health problems [10]. These are typically non-specific complaints, often with few biomedical findings and with a high rate of co-morbidity with other subjective health complaints [11–14]. The majority of sick leave episodes related to musculoskeletal and mental complaints are based on the patients’ subjective reports of pain and discomfort [15, 16]. Subjective health complaints have been suggested as a neutral term for these complaints [11, 17]. Common mental disorders, such as anxiety and depression, predict longer duration and higher recurrence of sick leave [18]. Multiple pain sites [19], higher levels of pain and discomfort, and more severe conditions have a negative effect on RTW and work disability [20, 21]. In addition to health complaints, a range of other factors has been found to predict non-RTW and disability after long-term sick leave. These factors include functional ability [22], beliefs and expectations about recovery and RTW [23], length of previous sick leave [7, 9, 14, 24], socioeconomic status [7, 8, 14, 25], and physical and psychosocial work factors [8, 20, 25]. With the exception of these findings, knowledge about predictive factors for continued sick leave after WR is limited, and there has recently been made a call for more refined research exploring indirect relationships between various psychosocial predictors of RTW [26].

**Fig. 1:**
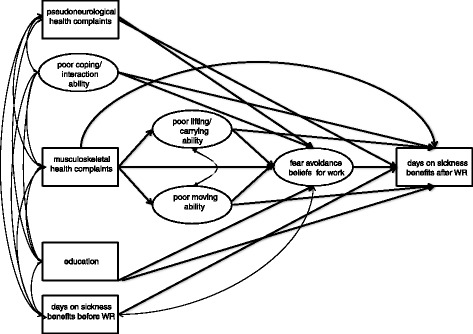
Hypothesized model: The circles represent latent variables and the squares represent observed variables. The latent variables are estimated by the use of the observed indicators described in the methods. For poor coping/interaction ability, the number of corresponding observed indicators was 8, for poor lifting/carrying ability it was 3, for poor moving ability it was 7, and for fear avoidance beliefs for work the number of indicators was 7. Double-headed slim arrows indicate correlations between independent variables

Fear avoidance beliefs are found to be a strong predictor for non-RTW among individuals with non-specific low back pain (LBP) [27–29]. However, relatively few studies have examined the predictive value solely of fear avoidance beliefs for work (FABW) on RTW. Due to the high rates of co-morbidity with other musculoskeletal and mental health complaints, it is reasonable to assume that the cognitive and behavioral predictors for RTW in individuals with LBP are applicable to other musculoskeletal conditions and to common mental health complaints [29]. We have earlier, in a similar Norwegian sample, shown that FABW were the strongest predictor for non-RTW at 3 and 12 months follow-up of WR participants [14]. The assessment of fear avoidance beliefs was originally based on a biopsychosocial model [30]. Fear avoidance beliefs are mediators between pain and avoidance behavior, such as sick leave and withdrawal from working life [31, 32]. Pain and avoidance behavior is determined by psychological processes in experience and interpretation of pain and discomfort [33, 34], and comprises sensory as well as cognitive, affective, behavioral, and social aspects [30, 35]. The meaning of pain to the individual depends on how the pain stimulus is evaluated, the expected outcome, based on previous experiences, and whether the individual expects to cope with the pain or not [36]. The Cognitive activation theory of stress (CATS) postulates that learned stimulus and response outcome expectancies determine psychobiological responses [36]. Individuals expecting to cope with a specific situation have established positive response outcome expectancy, while individuals who do not expect to cope may have negative response outcome expectancies (hopelessness) or no response outcome expectancies (helplessness) [36]. In the current study we propose and test five paths, and hypothesize that FABW will mediate the effects of subjective health complaints (musculoskeletal and pseudoneurological complaints), functional ability (poor coping/interaction ability, poor lifting/carrying ability and poor moving ability), and education on days on sickness benefits after WR (Fig. 1). We also hypothesized that high levels of earlier sick leave will lead to high levels after the intervention.

### Path 1

The path from musculoskeletal complaints via FABW to days on sickness benefits after WR (Fig. 1) is supported by previous research [27–29]. We hypothesize that the relation between musculoskeletal complaints and FABW can be explained by lowered physical function (moving ability and lifting/carrying ability) (Fig. 1). Some studies have found a negative relationship between musculoskeletal complaints and work-related functional abilities [37, 38], e.g. individuals on sick leave with musculoskeletal diagnoses report loss of physical function [37, 39]. However, how health complaints affect function in daily life and work depend on both individual and contextual factors [2, 40]. If an employee experiences pain and functional problems at the workplace while performing specific work tasks, he or she may avoid these tasks or avoid going to work at all. Avoiding the work tasks or the workplace can in certain cases be protective, and the employee learns that avoidance behavior is beneficial [32, 41]. Associative learning mechanisms can cause persistent workplace avoidance for a long time, even when there is no longer any risk of harm [41]. High levels of FABW may therefore lead to sustained avoidance behavior, and may be dysfunctional over time. We propose that the path from poor physical function to FABW goes from negative experiences to learned workplace avoidance, which in turn lead to negative response outcome expectancies towards going back to work.

### Path 2 and 3

The path from pseudoneurological complaints (i.e. tiredness, sadness/depression, anxiety) to FABW (Fig. 1) has previously been supported [14, 42, 43]. There is a strong association between psychological distress, such as depression and anxiety, and FABW, in individuals on sick leave with neck and back pain [42, 43]. Among WR participants, high level of pseudoneurological complaints explained a significant part of the variance in FABW [14]. Individuals on sick leave with common mental health disorders will typically report poor mental functioning often related to coping and interaction ability [37, 38]. Poor physical and mental functioning has been shown to be strongly associated with not returning to work 3 years after WR [44]. In the paths from pseudoneurological complaints and poor coping/interaction ability to continued sick leave, previously established negative response outcome expectancies may act as mediators in terms of FABW. They mediate between the stimulus, such as perceived psychosocial stress at the workplace, and the avoidance behavior e.g. not going to work [45].

### Path 4

We hypothesize that FABW will mediate, at least, some of the effect of level of education on non-RTW after WR. FABW are negatively correlated with education [46]. Education is often used as a proxy for socioeconomic status [47, 48], and is highly interrelated with occupational class and type of work [49, 50]. The level of education is strongly related to long-term sick leave [51] and non-RTW after WR [9, 14]. Individuals with lower education more often have physically demanding work with less control and decision latitude [48, 51]. Lower education is also associated with less psychosocial resources [52, 53], skills, and qualifications [54]. A discrepancy between work demands and available resources may lead to an enhanced stress response and to a feeling of helplessness and hopelessness, with biological and behavioral consequences [52, 53]. Loss of capacity to cope at work is therefore believed to trigger more fear and work avoidance behavior among individuals with low education.

### Path 5

Previous sick leave is a strong predictor of long-term sick leave and disability pension [7, 9, 14, 24]. One might hypothesize that FABW will mediate the effect of previous sick leave on RTW. However, there are to our knowledge, no current studies supporting a possible indirect effect of previous sick leave via FABW. Therefore, previous sick leave is included as an independent variable in the model, hypothesized to have a direct effect on days on sick leave during follow-up (Fig. 1).

The aim of the present paper was to test a mediation model for continued sick leave where we hypothesized that FABW would be an important mediator between known biopsychosocial predictors and the number of days on sickness benefits after WR. The model was tested using structural equation modeling (SEM) [55].

## Methods

### Participants

**Fig. 2:**
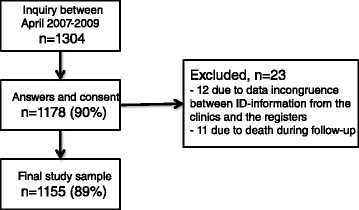
Study flowchart

This was a prospective cohort study with 1155 participants (69 % women) from eight different inpatient WR clinics in Norway (Study flowchart, Fig. 2). The participants were recruited between April 2007 and Mars 2009. Baseline characteristics are shown in Table 1.

**Table 1:** Baseline characteristics, means and standard deviation (SD). Women and men reported separately

	(*n* = 1155) [missing %]	Mean (SD) women (*n* = 806)	Men (*n* = 349)	*p*-value^#^
Age	46 (9.1) [0 %]	47 (8.9)	45 (9.4)	0.002*
Education	13 (2.9) [7.5 %]	13 (2.9)	12 (2.8)	<0.001**
Days on sickness benefits before WR	297 (189) [0 %]	292 (184)	307 (201)	0.234
Days on sickness benefits after WR	595 (424) [0 %]	590 (422)	607 (428)	0.536
Musculoskeletal complaints	9.5 (5.1) [1.3 %]	10.0 (5.0)	8.3 (4.6)	<0.001**
Pseudoneurological complaints	6.2 (3.9) [1.3 %]	6.60 (3.8)	5.3 (3.9)	<0.001**
Fear avoidance beliefs for work	23.1 (11.4) [7.8 %]	21.9 (11.5)	26.1 (10.8)	<0.001**
Moving ability	1.5 (0.5) [0.2 %]	1.5 (0.5)	1.7 (0.5)	<0.001**
Lifting/carrying ability	1.6 (0.6) [0.4]	1.6 (0.6)	1.6 (0.6)	0.392
Coping/interaction ability	1.7 (0.5) [0.8 %]	1.7 (0.5)	1.6 (0.5)	0.055

^#^Independent samples T-tests, **p < 0.001, *p < 0.01

### Procedure

At arrival to the WR clinic, all patients were gathered to an information meeting. Oral and written information was given according to the Declaration of Helsinki, with information about study aims and procedures, and assurance that withdrawal was possible at any time without any consequences for the treatment. All participants who returned a written informed consent and who answered a comprehensive questionnaire were included in the study. There were no further exclusion criteria. The participants were followed with register data for 3 years and 4 months (1217 days). The follow-up data on sickness benefits were obtained from official registers from The Norwegian Labour and Welfare Administration (NAV) in July 2012. The study fulfilled the principles in the Declaration of Helsinki, and was approved by the Medical Ethics Committee; Region West in Norway (REK-vest ID 3.2007.178) and the Norwegian social science data services (NSD, ID 16139).

### Work rehabilitation

All the participants completed inpatient WR programs administrated within the specialized health care in Norway. Patients were admitted to WR mainly based on referrals from their general practitioners. Target groups of the rehabilitation programs were individuals on long-term sick leave, typically with diagnoses related to musculoskeletal and/or mental health complaints. The goal of the WR programs was to improve the level of functioning, enhance work ability, and increase the likelihood of RTW. The content of the WR programs were similar, but the length of the programs varied from 3 to 6 weeks with a mean length of 31 days (SD = 11). The programs were run by interdisciplinary rehabilitation teams, constituted by at least four of the following professions; physicians, work consultants, nurses, physiotherapists, sport pedagogues, and occupational therapists. The content of the programs included a combination of individual and group based interventions with physical activity, education, cognitive behavioral modification, and cooperation with relevant stakeholders. One clinic offered work training in different manual workshops, amounting to one third of the rehabilitation program. At the end of the WR program, a follow-up plan was developed together with the participant, with RTW as the main goal. This plan could include future participation from several stakeholders outside the WR setting, e.g. different health care providers, the workplace, or the local social insurance office.

### The Norwegian sickness insurance system

An employee is entitled to sickness benefits (sick leave benefit, work assessment allowance, or disability benefit) from NAV if incapable of working due to disease or injury. From the first day of reporting sick and up to 1 year, an employee is entitled to a sick leave benefit equal to 100 % of their regular salary in compensation from the first day of reported sick. The sick leave benefit can be partial and graded from 20 to 99 %. If the employee does not return to work after 1 year, the employee may receive a work assessment allowance (WAA), which has an upper limit of 4 years. A WAA is granted for individuals going through medical treatment or rehabilitation, or individuals that might benefit from vocational rehabilitation actions to RTW. If the employee does not return to work after fulfilled WAA, a disability pension (DP) may be granted to individuals with permanent incapacity for work, defined as having work ability reduced by at least 50 %. As a main rule, WAA and DP constitute 66 % of the salary the last year as an employee.

### Measures and instruments

All the predictor variables; education, days on previous sickness benefits, health complaints, functional ability, and fear avoidance beliefs for work, were measured at baseline. Days on sickness benefits before and after WR were obtained from official registers from NAV, and were adjusted for receiving partial benefits. Partial benefits were adjusted so that 50 % sick leave was registered as half a working day. Overlap between start and end date for the registered sickness benefits could occur in the registers when the person moved from one type of benefit to another. To avoid double counting, we let the new benefit replace the old.

#### Outcome measure

Continued sick leave was measured as the total number of days on registered sickness benefits during the follow-up period of 3 years and 4 months.

#### Predictor variables

- Education was measured by a single question about total completed years of schooling/studies, counted from the first year of primary/elementary school.
- Previous sick leave was measured as the total days on registered sickness benefits during the last 2 years before entry to the WR program, prior to entering the study.

### The subjective health complaints (SHC) inventory [11]

Two subscales from the SHC-Inventory measured musculoskeletal and pseudoneurological complaints. These two scales were utilized as they represent the most common complaints among musculoskeletal and mental complaints, causing sick leave [16]. Intensity of each complaint is scored on a four-point scale from 0–3, where 0 is no complaints and three is severe complaints. Predictive validity of the subscales has previously been reported [11, 56].

1. *“Musculoskeletal complaints”, 8* items, (shoulder pain, neck pain, upper back pain, arm pain, headache, low back pain, leg pain during physical activity, migraine).
2. *“Pseudoneurological complaints*”, 7 items, (tiredness, anxiety, sleep problems, sadness/depression, dizziness, heat flushes, extra heartbeats).

### The Norwegian Function Assessment Scale (NFAS) [37, 38]

Physical and mental function during the last week were measured with the NFAS, rated on a four-point scale from 1–4, where one is no functional limitations and four is cannot perform [37, 38]. The original scale consists of 39 items and seven domains, and has been shown to be a valid instrument for evaluation of work-related function in a previous Norwegian study [37]. Three new subscales derived from the NFAS were used, measuring physical function and coping/interaction ability. The new scale consists of 18 items and three factors (see statistical methods).

1. *“Moving ability”, 7* items, α = 0.83 (standing, walking more than a km on flat ground, walking on different surfaces, putting on your shoes and socks, dressing and undressing, cleaning your house, sitting on a kitchen chair).
2. *“Lifting/carrying ability”, 3* items, α = 0.75 (carrying shopping bags in your hands, carrying a little sack/backpack on your shoulders or back, pushing and pulling with your arms).
3. *“Coping/interaction ability”,* 8 items, α = 0.79 (staying alert and being able to concentrate, working in groups, guiding others in their activities, managing everyday responsibility, managing everyday stress and strains, managing to take criticism, managing to control your anger and aggression, remembering things).

### The Fear Avoidance Beliefs Questionnaire (FABQ) [30]

FABW were measured using the FABQ-Work subscale from the FABQ. Each item is rated on a seven-point Likert scale ranging from 0–6, where 0 is completely disagree and 6 is completely agree. Good reliability and construct validity have been reported [30, 57].

*“Fear avoidance beliefs for work”*, 7 items, α = 0.87 (My pain was caused by my work or by an accident at work. My work aggravated my pain. My work is too heavy for me. My work makes or would make my pain worse. My work might harm me. I should not do my normal work with my present pain. I do not think I will be back to my normal work within 3 months).

The questionnaire was slightly modified to concern individuals with pain in general, and not only back pain. Introductorily one question was added, asking whether the respondents were bothered with pain or not, and it was followed by a multiple response question on pain location (back, shoulder/arm, neck, leg/feet, head, chest or other).

#### Statistical methods

Baseline characteristics were examined using SPSS statistics version 21 for Windows. Differences in socio demographic and questionnaire data between genders were examined by Chi square tests (*x*^2^) in non-parametric data, and independent samples t-tests in parametric data.

### Data handling

Performing a confirmatory factor analysis (CFA) of the original scale of NFAS [37, 38] did not confirm the original factor structure of seven domains. Therefore an exploratory factor analysis (EFA) was performed, using the robust-weighted least square estimator (WLSMV) and geomin oblique rotation. The EFA was conducted in Mplus, allowing for categorical items. The EFA revealed the presence of three factors. Twenty-one items were removed from the three factors to create meaningful entities and to avoid cross loadings. The subsequent EFA on the remaining 18 items supported the structure of the same three factors. Chronbach’s alpha (α) was used to determine the internal consistency of the three subscales based on the derived factors: 1) *“Moving ability”, 7* items, α = 0.83. 2) *“Lifting/carrying ability*”, 3 items, α = 0.75. 3) *“Coping/interaction ability”,* 8 items, α = 0.79.

### Structural equation modeling

The hypothesized model was tested using structural equation modeling (SEM) [55]. SEM is a multivariate technique, which combines path analysis and measurement (factor) models [55]. SEM may combine observed and latent variables, and is a confirmatory technique where SEM is used to determine if the a priori model is supported by the data [55]. The SEM analyses were performed with Mplus version 7.00 program package [58] using the robust-weighted least square estimator (WLSMV). The WLSMV estimator was used because all of the indicators of the latent variables were treated as ordinal. WLSMV uses polychoric correlations for estimation, seems relatively robust to violations of normality [59, 60], and provides consistent estimates when missing data are random with respect to the covariates in the model [61]. The Comparative Fit Index (CFI) and the Root Mean Square Error of Approximation (RMSEA) were used to assess model fit as recommended by Brown [60]. A CFI between 0.90 and 0.95 indicates a fair model fit, with values above 0.95 to be a good fit, and a RMSEA less than 0.08 indicates a fair model fit, with values below 0.05 to be a good fit, between the measurement model and the observed data [60].

The structural measurement model was estimated with number of days on sickness benefits after WR as an observed dependent variable. Education and number of previous days on sickness benefits were treated as observed variables as they were both based on a single item. Musculoskeletal complaints and pseudoneurological complaints were also treated as observed variables, because their associated items were considered as formative/causal indicators and not as reflective of a common factor [55]. FABW and the three subscales of functional ability; coping/interaction ability, lifting/carrying ability, and moving ability, were treated as latent variables in the model as their associated items were assumed to be caused by underlying common factors [55].

The hypothesized SEM model (Fig. 1) was tested by the use of a two-step modeling approach [55]. In the first step, we tested the adequacy of the measurement models. The hypothesized three-factor model derived from the NFAS (moving ability, lifting/carrying ability, and coping/interaction ability) and the hypothesized unidimensional FABW model were tested separately. To identify sources of misfit in potentially inadequately fitting measurement models, modification indices were inspected [62]. In the second step the adequacy of the full structural regression model was tested, and the significance of indirect effects was tested by the use of the Sobel (delta) method [58].

**Fig. 3:**
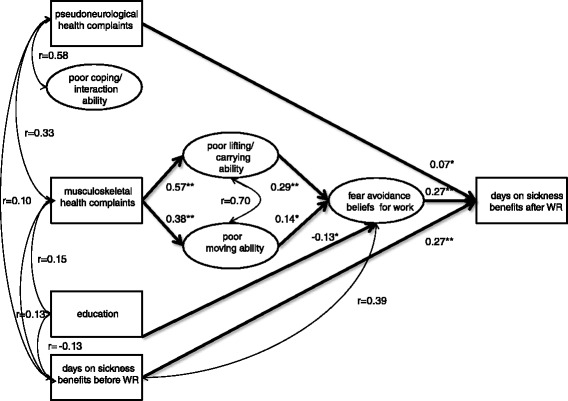
Parameter estimates for the final model. The circles represent latent variables. The squares represent observed variables. Double-headed slim arrows indicate correlations between independent variables. Non-significant paths/correlations are not shown. Model fit: (*x*2 [370] = 1409,335, *p* < 0.001, CFI = 0.957, RMSEA = 0.049 (90 % CI: 0.046–0.052) **p < 0.001, *p < 0.05

Multiple group analyses [62, 63] were used to test whether the model was invariant across gender. When testing whether the measurement model was invariant across gender, each latent construct was tested separately. In these analyses a top down strategy was applied [58] where the fit of a model of which the loadings and thresholds were held equal between genders was compared to a model of which the same parameters (except for the identification item) were free to vary. The model was assumed non-invariant if the change in chi square was significant (tested by DIFFTEST in Mplus) and the decrease in CFI was less than 0.002 [55, 64]. Only the DIFFTEST procedure was used to test whether the paths and correlations in the structural model were invariant across gender. In the final multiple group analysis the paths that were significantly different between men and women were estimated freely, while the non-significant paths were set equal between men and women. Direct and indirect effects were estimated as indicated in Fig. 3. Standardized estimates and p-values were reported.

## Results

All participants were either on partial or fulltime sickness benefits when they were admitted to the WR program. Mean time on sickness benefits during the last 2 years before admittance to the WR program, were 10 months (SD = 6.7). Baseline characteristics are shown in Table 1.

### Descriptive statistics

During the follow-up period, the participants received sickness benefits for an average of 595 (SD = 424) days. There were no significant gender differences in days on sickness benefits before or after the WR program (Table 1). Significant gender differences were found for age, education, SHC, FABW, and functional ability. Men were significantly younger, reported fewer years of education, less severe SHC, higher levels of FABW, and poorer moving ability. Women reported poorer coping/interaction ability (Table 1). On the modified version of the FABQ, 95 % of the women and 91 % of the men reported having pain, and a majority reported several pain sites. In terms of pain location, a statistically significant higher proportion of the women reported pain in shoulder/arm, neck, head, and other pain sites (Chi-square test, all *p* < 0.001).

### Correlations

Correlations based on the sum scores of the different scales are shown in Table 2. The correlations between the observed variables were of small to moderate magnitude and most were as expected by our model. Days on sickness benefits after WR were significantly correlated with all the included variables in the expected direction (range *r* = -0.12–0.39). The correlation between days on sickness benefits after WR was most pronounced with FABW (*r* = 0.38) and previous days on sickness benefits (*r* = 0.39). Given the prominent place of FABW in our model, it was not surprising that it was significantly correlated with most of the other variables. It was surprising, however, that FABW was neither significantly correlated with pseudoneurological complaints (*r* = 0.04, *p* > 0.05) nor with coping/interaction ability (*r* = 0.02, *p* > 0.05).

**Table 2:** Correlations between days on sickness benefits before WR, education, subjective health complaints, functional ability, fear avoidance beliefs for work and days on sickness benefits after WR

		1	2	3	4	5	6	7	8
1	Days on sickness benefits before WR	-							
2	Education	−0.13**							
3	Musculoskeletal complaints	0.13**	−0.15**						
4	Pseudoneurological complaints	0.10**	0.13**	0.33**					
5	Moving ability	0.19**	−0.25**	0.41**	0.03				
6	Coping/interaction ability	0.10*	0.12**	0.13**	0.58**	0.04			
7	Lifting/carrying ability	0.22**	−0.24**	0.57**	0.15**	0.70**	0.15**		
8	Fear avoidance beliefs for work	0.33**	−0.25**	0.29**	0.03	0.40**	0.02	0.45**	
9	Days on sickness benefits after WR	0.39**	−0.12**	0.15**	0.13**	0.22**	0.13**	0.23**	0.38**

**p <0.001, *p <0.01

### SEM analyses

#### Gender differences

Preliminary multigroup analyses on all the included models showed some gender differences in the structural parameters. When testing for measurement invariance across gender for each latent construct separately, the analyses revealed strong measurement invariance for coping/interaction ability and lifting/carrying ability, and partial measurement invariance for FABW and moving ability. More specifically, given equal trait levels of FABW across gender, men had a higher score on the following items: “My pain was caused by my work or by an accident at work”, “My work might harm me”, and “I should not do my normal work with my present pain”. Women were more likely to report that they had more problems “cleaning your house” than men with an equal level of moving ability. Multigroup analyses on the full structural model furthermore revealed that education was significantly correlated only with pseudoneurological complaints (*r* = 0.31 vs. *r* = 0.03) and coping/interaction (*r* = 0.26 vs. *r* = 0.05) amongst men. Most importantly however, no significant differences were found between genders on the structural paths in the full model. Men and women were therefore treated as one group in the following analyses and results presented.

#### Step 1: CFA measurement models

Neither the hypothesized three-factor model derived from the NFAS (*x*^2^ [132] = 1232.962, *p* < 0.001, CFI = 0.926, RMSEA = 0.085, 90 % CI for RMSEA = 0.081–0.089) nor the hypothesized unidimensional FABW model (*x*^2^ [14] = 260.797, *p* < 0.001, CFI = 0.978, RMSEA = 0.128, 90 % CI for RMSEA = 0.115–0.142) had an adequate fit to the data. For functional ability, the three-factor solution had an acceptable fit (*x*^2^ [130] = 755.998, *p* < 0.001, CFI = 0.958, RMSEA = 0.065, 90 % CI for RMSEA = 0.060–0.069) when allowing local dependencies (correlated error terms) between the items “putting on your shoes and socks” and “dressing and undressing” (*r* = 0.74), and between the items “walking more than a km on flat ground” and “walking on different surfaces” (*r* = 0.67). Both of these local dependencies were located on the moving factor. For FABW, the model fit indices for a one-factor solution was acceptable (*x*^2^ [11] = 62.381, *p* < 0.001, CFI = 0.995, RMSEA = 0.066, 90 % CI for RMSEA = 0.051–0.082), after allowing local dependencies between: “I do not think I will be back to my normal work within 3 months” and “I should not do my normal work with my present pain” (*r* = 0.30), “My work makes or would make my pain worse” and “My work aggravated my pain” (*r* = 0.42), and “My work aggravated my pain” and “My pain was caused by my work or by an accident at work” (*r* = 0.30). Even if the hypothesized measurement models had to be modified somewhat, it can be argued that the results supported the construct validity of the latent constructs as all the items had rather high loadings on their respective latent variables (standardized loadings for moving ability ranged between 0.66 and 0.81; FABW ranged between 0.54 and 0.88) which supports that these constructs are essentially unidimensional despite some local dependencies.

#### Step 2: The full structural model

The full structural model had a good fit to the data (*x*^2^ [370] = 1409,335, *p* < 0.001, CFI = 0.957, RMSEA = 0.049, 90 % CI for RMSEA = 0.046–0.052). The analyses supported the hypothesized important role of both FABW (Standardized Beta = 0.27, *p* < 0.001) and days on sickness benefits before WR (Standardized Beta = 0.27, *p* < 0.001) in predicting days on sickness benefits after WR (Fig. 3). Also as hypothesized, FABW mediated the paths between both education (Standardized Beta for indirect effect = -0.034, *p* < 0.01) and musculoskeletal complaints on days on sickness benefits after WR. As predicted the latter mediation effect went via two different routes. One of the indirect effects went from musculoskeletal complaints to days on sickness benefits after WR via lifting/carrying ability (Standardized Beta for indirect effect = 0.045, *p* < 0.001). The other indirect effect went via moving ability (Standardized Beta for indirect effect = 0.015, *p* < 0.05) prior to going via FABW (Fig. 3). The indirect effects from both poor lifting/carrying ability (Standardized Beta for indirect effect = 0.08, *p* < 0.001) and poor moving ability (Standardized Beta for indirect effect = 0.039, *p* < 0.05) via FABW were significant. Poor coping/interaction ability did not have a direct or an indirect effect on days on sickness benefits after WR. Pseudoneurological complaints had only a very weak direct effect (Standardized Beta = 0.07, *p* < 0.05) on days on sickness benefits after WR.

## Discussion

The results partly supported our main hypothesis, which stated that FABW are an important mediator between the predictors; health complaints, functional ability, and socioeconomic status, and the outcome; continued sickness benefits 3 years after WR. FABW seem to mediate the effect of physical function and level of education on days on sickness benefits after WR. Also, as hypothesized, musculoskeletal complaints had an indirect effect on continued sick leave via physical function and via FABW. The present analyses did not support the hypothesis that pseudoneurological complaints or poor coping/interaction ability lead to continued sick leave after WR via FABW. Whereas pseudoneurological complaints only had a small direct effect on days on sickness benefits after WR, poor coping/interaction ability did not predict either FABW or days on sickness benefits after WR. There were no gender differences in the mediation model, indicating that the factors involved in the process of RTW after long-term sick leave and WR may be equal for men and women.

A key message from this study is that FABW are a mediator between various predictors and continued sickness benefits after WR in individuals with long-lasting musculoskeletal complaints and multiple pain sites. Most of the studies showing that cognitions and beliefs predict work outcomes have been on individuals with LBP [29], but results are weak and inconsistent for fear avoidance beliefs predicting RTW in samples of individuals with chronic LBP [27]. Our results are in line with previous findings showing that FABW was a main predictor of non-RTW at 3 and 12 months follow-ups after WR [14].

This study adds to the literature by showing direct and indirect relationships between various predictors and FABW and continued sick leave after WR, in a predefined mediation model. FABW is a complex phenomenon, shaped in the interplay between internal and external stressors, from competing personal goals, psychosocial factors, and daily life and workplace factors [32]. In individuals on sick leave with long-lasting health complaints, the internal stressors may be related to the perception of pain, distress and functional ability, and the external stressors to perceived stress and discomfort at the workplace. Fear avoidance beliefs are linked to avoidance behavior, and may act as a mediator between the internal and external stressors and avoiding the workplace [45]. Stimulus expectancies and learned positive or negative response outcome expectancies as described in the CATS determine psychobiological responses [55]. High levels of FABW and subsequent avoidance behavior can be explained as negative response outcome expectancies towards RTW, e.g. poor coping.

Our results revealed no direct path from musculoskeletal complaints to days on sickness benefits after WR. This finding support the understanding that biomedical factors do not directly influence RTW after long-term sick leave, but rather work indirectly through other factors such as functional ability and beliefs [3, 29, 65]. Likewise, we believe that the strong indirect effects found for musculoskeletal complaints via physical function and further via FABW to continued sick leave after WR, support the use of a biopsychosocial approach when predicting RTW after long-term sick leave and rehabilitation efforts [3, 4, 65]. The paths from musculoskeletal complaints to poor physical function as measured by moving ability and lifting/carrying ability, were strong, and in line with previous research [37–39]. Functional limitations may be superior to pain for predicting disability outcomes and RTW [66].

The results supported our hypothesis of a path from level of education via FABW to continued sickness benefits after WR. Individuals with low education have more often manual and physically demanding work with less control and decision latitude [48, 49]. This may lead to high levels of negative workplace exposures [49] and FABW. Individuals with low level of education may also have less psychosocial resources to deal with the work demands [52, 53]. Consequently, there may be a discrepancy between demands and available resources, which in turn may cause high activation, negative outcome expectancies, and prolonged workplace avoidance in terms of prolonged sick leave.

Another main finding was that length of previous sick leave at admittance to the WR program had a direct effect on days on sickness benefits after WR. There are strong indicators for negative and independent relationships between length of previous sick leave and the probabilities for returning to work [7, 9, 14, 24]. However, one might also assume an indirect effect of previous sick leave via FABW. This issue should be addressed in future research.

For pseudoneurological complaints, the results did not support the hypothesis of FABW being a mediator of continued sick leave after WR. This result is purely a consequence of the very small correlations between FABW and pseudoneurological complaints in our data. However, this finding is surprising, since previous results in a similar study population of long-term sick-listed WR participants, found pseudoneurological complaints to explain a significant part of the variance in FABW [14]. Similarly, in individuals on sick leave due to neck and back pain, there were a strong relationship between psychological distress, such as depression and anxiety and FABW [42, 43]. A possible explanation of the lack of association between pseudoneurological complaints and FABW in this study may be that the current study population, from eight different WR clinics, is more heterogeneous than the previous study population of WR participants [14]. This may imply a higher variance in reports on the musculoskeletal and pseudoneurological variables, and less overlap between these complaints. An argument against this explanation is however, that pseudoneurological complaints in the current study had a direct predictive effect on continued sick leave after WR, in line with findings in the previous study where pseudoneurological complaints predicted work outcomes 3 months after participating in WR [14]. When the fear avoidance beliefs questionnaire was developed, it was strongly emphasized that there was an affective dimension, in the form of anxiety, high somatic awareness, and depressive symptoms, between maladaptive beliefs and developing chronic pain [30]. The associations and mechanisms between common mental complaints, FABW, and RTW seem still poorly understood in sick-listed WR participants with long-lasting composite health complaints.

Furthermore, the results did not support our hypothesis of an effect of coping/interacting ability on continued sick leave benefits after WR, neither directly nor indirectly via FABW. This lack of association is also reflected by the very small correlation with FABW in this data set. In a previous study, we found that individuals on sickness benefits 3 years after WR reported poorer physical and mental functional ability than those who had returned to work [44]. Functional ability is dependent on the situation, as the capacity of an individual always will be restricted or facilitated in interacting with contextual factors, like the work environment [40]. We did however not include any work-related variables in the current SEM model. More research investigating direct and indirect relationships between individual psychosocial factors and environmental workplace factors is needed to understand more of what facilitates and hinders RTW in individuals on sick leave [3, 26].

The large and representative study sample of WR participants from eight different rehabilitation clinics in Norway is a strength in the present study. A multicenter sample may give a more heterogeneous study population. Heterogeneity may give high generalizability when the prognostic model matches the observed outcome [67]. Access to complete official register data and the long follow-up period of sickness benefits strengthen the interpretation of the results. A limitation may however be that all variables expect the outcome measure were collected at entry to the WR program. This clearly limits causal interpretations between the constructs. Longitudinal studies focusing on change in the constructs included in the model have been recommended [26], and should be a priority in later studies. A limitation may also be that the WR program could influence some of the included independent variables, related to health, functioning, and FABW, and they may change during follow-up. This potential bias will however be equal for all the participants. In this paper, we choose to include the independent factors measured at baseline, because we were interested in the prediction effect and not the changes over time. Future studies might explore if any changes in these variables during or after the rehabilitation will be stronger predictors for RTW after WR.

In our final model, poor coping/interaction ability was not a significant predictor for continued days on sickness benefit. This may be due to its high correlation with pseudoneurological complaints. A potential interesting hypothesis for future research is that pseudoneurological complaints may mediate the relation between poor coping/interaction ability and continued days on sickness benefit. Although the data partly supported the hypothesized mediation model, the estimates for the single pathways were not very strong. It is therefore important to identify other predictors and pathways intervening with education, health complaints, functional ability, and fear avoidance beliefs for work. Research should in particular address how individual factors intervene with contextual factors, e.g. at the workplace. In addition, using measures on work exposure or work environmental factors in our model could have given a stronger design, making it possible to adjust for possible contextual confounders. Despite these limitations, the results from this study may have implications for the process of referral to WR programs and for determining the content of the programs. Our results suggest that clinicians and stakeholders should have an increased focus on individuals with high levels of FABW and poor physical function among those reporting musculoskeletal complaints, and on the severity of complaints among those reporting pseudoneurological complaints. For individuals at risk, increased attention should be on the workplace, in particular on work tasks and the organization of work, for instance via improved learning climate and learning opportunities [52].

## Conclusions

The hypothesized model was partly supported by the data. The results show that FABW may mediate the effect of musculoskeletal complaints via physical function, and the effect of education on continued sickness benefits 3 years after participating in a WR program. These findings may give direction for future research assessing prognostic factors for RTW outcomes after long-term sick leave in individuals with long-lasting health complaints.

## Abbreviations

CATS: the cognitive activation theory of stress
CFA: confirmatory factor analysis
CFI: the comparative fit index
CI: confidence interval
DP: disability pension
EFA: exploratory factor analysis
FABQ: the fear avoidance beliefs questionnaire
FABW: fear avoidance beliefs for work
LBP: low back pain
NAV: The Norwegian Labour and Welfare Administration
NFAS: The Norwegian function assessment scale
NSD: The Norwegian social science data services
REK: Region Medical Ethics Committee
RMSEA: the root mean square error of approximation
RTW: return to work
SD: standard deviation
SEM: structural equation modeling
SHC: subjective health complaints
WAA: work assessment allowance
WLSMV: the robust-weighted least square estimator
WR: work rehabilitation
